# Repeat Age Decomposition Informs an Ancient Set of Repeats Associated With Coleoid Cephalopod Divergence

**DOI:** 10.3389/fgene.2022.793734

**Published:** 2022-03-14

**Authors:** Alba Marino, Alena Kizenko, Wai Yee Wong, Fabrizio Ghiselli, Oleg Simakov

**Affiliations:** ^1^ Department for Neurosciences and Developmental Biology, University of Vienna, Vienna, Austria; ^2^ Department of Biological, Geological, and Environmental Sciences, University of Bologna, Bologna, Italy; ^3^ Institute of Evolutionary Science of Montpellier, University of Montpellier, Montpellier, France

**Keywords:** cephalopods, genome architecture, evolution, repeat elements, LINEs, SINEs, ancient repeat complement

## Abstract

In comparison with other molluscs and bilaterians, the genomes of coleoid cephalopods (squid, cuttlefish, and octopus) sequenced so far show remarkably different genomic organization that presumably marked the early evolution of this taxon. The main driver behind this genomic rearrangement remains unclear. About half of the genome content in coleoids is known to consist of repeat elements; since selfish DNA is one of the powerful drivers of genome evolution, its pervasiveness could be intertwined with the emergence of cephalopod-specific genomic signatures and could have played an important role in the reorganization of the cephalopod genome architecture. However, due to abundant species-specific repeat expansions, it has not been possible so far to identify the ancient shared set of repeats associated with coleoid divergence. By means of an extensive repeat element re-evaluation and annotation combined with network sequence divergence approaches, we are able to identify and characterize the ancient repeat complement shared by at least four coleoid cephalopod species. Surprisingly, instead of the most abundant elements present in extant genomes, lower-copy-number DNA and retroelements were most associated with ancient coleoid radiation. Furthermore, evolutionary analysis of some of the most abundant families shared in *Octopus bimaculoides* and *Euprymna scolopes* disclosed within-family patterns of large species-specific expansions while also identifying a smaller shared expansion in the coleoid ancestor. Our study thus reveals the apomorphic nature of retroelement expansion in octopus and a conserved complement composed of several DNA element types and fewer LINE families.

## Introduction

Coleoid cephalopods (squid, cuttlefish, and octopus) are characterized by a highly derived body plan compared to the other molluscs, with the main novelties being a partial or complete loss of the shell, a crown of flexible arms provided with suckers (Boletzky, 2003), camera-type eyes, and a nervous system considered to be the most complex among invertebrates (Young, 1963). Such phenotypic features are further closely related to the active predatory lifestyle and the wide variety of behaviors in extant cephalopods (Hanlon and Messenger, 2018). In recent years, cephalopods emerged as intriguing organisms in the genome evolution field as they showcase several types of genomic features, including rearrangements of bilaterian-conserved local gene linkages, gene duplications, orphan gene emergence, and repeat element expansions (Albertin et al., 2015; Belcaid et al., 2019). These signatures at different levels of genome organization were associated with the evolution of distinct organs within a single organism (Belcaid et al., 2019) and are likely to have co-evolved, comprising a complex evolutionary genome signature that ultimately contributed to the phenotypic novelties of cephalopods (Ritschard et al., 2019). Even though transposable elements (TEs) were initially classified as “junk” (Ohno, 1972) or “selfish” DNA (Doolittle and Sapienza, 1980; Orgel and Crick, 1980), their role as important mutation sources and therefore as determinants in the evolution of their hosts is now established. Indeed, depending on the target and mode of their transposition and recombination, mobile elements can be exapted to new *cis*-regulatory elements (Britten, 1996; Marino-Ramirez et al., 2005), disrupt or rewire regulatory networks (Feschotte, 2008; Moschetti et al., 2020; Sundaram and Wysocka, 2020), and cause chromosomal-level rearrangements (Gray, 2000). Besides, TEs are important tools for the development of new genomic integration (Sandoval-Villegas et al., 2021) and expression vector technologies (Palazzo and Marsano, 2021). TEs are present in every eukaryotic genome in very different proportions and classes (Wells and Feschotte, 2020), with both random drift and natural selection contributing to their differential amplification in divergent lineages (Lynch and Conery, 2003; Kent et al., 2017). About half of every sequenced coleoid cephalopod genome comprises repetitive DNA, whose composition significantly differs across lineages: SINEs are the main components of *Octopus bimaculoides* and *O. vulgaris* transposomes; LINEs prevail in *O. minor* and *Euprymna scolopes*, whereas mostly DNA elements are present in the *Architeuthis dux* genome (Albertin et al., 2015; Kim et al., 2018; Belcaid et al., 2019; Zarrella et al., 2019; Fonseca et al., 2020). Unlike coleoids, the *Nautilus pompilius* genome is smaller, is less repetitive (31%), and lacks the many genomic features of coleoid cephalopods (Zhang et al., 2021). Despite no functional survey being available, TEs are found to be extensively expressed in *O. bimaculoides* and *O. vulgaris* tissues (Albertin et al., 2015; Petrosino et al., 2021); furthermore, regions nearby loci that underwent rearrangements in coleoid cephalopods are rich in repeats in *O. bimaculoides*, just as orphan genes associated with novel structures are in *E. scolopes* (Albertin et al., 2015; Belcaid et al., 2019; Petrosino et al., 2021). Such observations highlight the central role that TEs might have played in cephalopod diversification. Although many of the repeat families have expanded recently in individual lineages, their role in shaping the ancestral coleoid cephalopod genome remains elusive. Furthermore, information about repeats in mollusks is fragmented as it is not usually presented with a wide comparative purpose (Zhang et al., 2012; Simakov et al., 2013; Wang et al., 2017; Powell et al., 2018; Kenny et al., 2020; Zeng et al., 2020); additionally, the number of sequenced cephalopod species is scarce. This hinders the systematic comparison of TE content within a clade, making it hard to have an overview of the present and past cephalopod repeat landscape. Our study aims to make a first step in this direction by providing a common repeat annotation of the main cephalopod lineages and extrapolating with a comparative approach the ancient TE landscape that possibly existed in the stem coleoid lineage. To this end, we considered the genome assemblies of the coleoids *O. vulgaris*, *O. bimaculoides*, *A. dux*, *E. scolopes*, and *N. pompilius*. Octopuses’ common ancestor dates back to ∼25 Mya (Uribe and Zardoya, 2017) and that of coleoids dates back to ∼270 Mya (Tanner et al., 2017), while *Nautilus* lineage diverged ∼415 Mya from coleoids (Bergmann et al., 2006; Kröger et al., 2011). We characterized both the total and divergence-based repeat contents in every species. Based on sequence divergence, we identified shared ancient TE families present across coleoid genomes. Finally, using sequence similarity network approaches, we could reveal complements of closely related squid and octopus sequences among the most abundant TE families, possibly hinting at their common origin back in the coleoid lineage.

## Methods

We used the scaffold-level genome assemblies of *O. vulgaris*, *O. bimaculoides*, *A. dux*, and *N. pompilius*, publicly available under GenBank accession numbers GCA_003957725.1, GCA_001194135.1, GCA_006491835.1, and GCA_018389105.1, respectively. A chromosomal-scale assembly generated with LACHESIS (Burton et al., 2013) was used for *E. scolopes* (Schmidbaur et al., in review, http://metazoa.csb.univie.ac.at/data/v2/). Completeness of genomes was assessed with BUSCO 5.2.2 (Manni et al., 2021) by considering the 954 conserved orthologs of the metazoa_odb10 database and with technical statistics supplied by Quast 5.0.2 (Gurevich et al., 2013) (Supplementary Table S1). For each assembly, the same repeat annotation workflow was employed: a family library was generated with RepeatModeler 2.0 (Flynn et al., 2020) and used to annotate and mask each starting assembly with RepeatMasker 4.0.9 (Smit et al., 2020); in order to uncover further sequences that were not detected in the first masking round, these steps were performed a second time on the previously hard-masked genome (double-masking, as employed in Meyer et al., 2021); a defragmentation step of all the obtained sequences was then carried out with RepeatCraft in the “strict” merge mode (Wong and Simakov, 2019).

Custom Bash, Python, and R scripts were used to filter and parse the data for the assessment of repeat content. Because the “Unknown” and “Simple_repeat” categories constituted a significant portion of the total repeats (see Supplementary Table S1) but were not of interest for our purpose, they were discarded to obtain a clearer landscape of the known TEs. Any repeat content that is henceforth referred to is therefore intended as deprived of unknown and simple repeats. Total repeat composition was assessed for every assembly in terms of subclass and family raw counts. Such content was then split into three contiguous intervals of divergence from consensus, namely, 0–10, 10–30, and >30%, as defined by RepeatMasker estimation with the Kimura distance-based method. We then looked for expression evidence by comparing RNA-seq data from different tissues with the repeat annotations to have an overview of the repeat complement activity of every species, except *A. dux*, for which transcriptomic data are not available (for data accessions, see Supplementary Table S3). After adapter and quality trimming (TrimGalore 0.6.5, Krueger, 2015), the reads were mapped to their genome with Hisat2 2.1.0 (Kim et al., 2019) and their coordinates were intersected with the repeat annotations in bedtools 2.29.2 with an overlap of 100% for the repeat sequences (Quinlan & Hall, 2010). Regardless of the expression pattern, weighted TE family composition in every bin, both with all families and with only shared families, was used to estimate Euclidean distances between species and carry out a principal component analysis (PCA). An “ancient” repeat subset was extracted by retaining only TE families represented in the >30% bin of every species. A 30% cutoff was chosen to identify old repeat copies as this distance is close to the RepeatMasker distance detection limit (around 50%): indeed, 5% maximum of the total elements was detected beyond this distance, and even fewer elements were found above 40% divergence (Supplementary Table S1). Such a complement was further characterized in *O. bimaculoides* and *E. scolopes*. For each family, the relationship between raw repeat counts per chromosome and chromosome sizes was estimated in *E. scolopes*. Finally, octopus and Hawaiian bobtail squid sequences of all divergence values from some of the most abundant families—CR1, RTE-BovB, Dong-R4, Penelope, and TcMar-Tc1—were compared with blastn from ncbiblast+ 2.10.0 with search options -task blastn and -word_size 18 (Altschul et al., 1990). A distance calculated as the number of mismatches/alignment length was assigned to each pairwise hit and used to resolve intra- and inter-species relations within each TE family. The R packages igraph, ggplot2, RcolorBrewer, and plyr were used for graphically representing the distances. Since the overall repeats were too many to be handled by R, the entire set of sequences in a bin was retained when possible, but in most cases, a downsampling of 1% or 10% was applied to obtain a readable graph. In addition to this distance-based network approach, we looked for homologies between cephalopod repeats and sequences of distantly related taxa that could hint at potential horizontal gene-transfer events (HGT) underlying cephalopod repeat bursts. To do this, we conducted BLAST searches of the TE family consensi in Dfam 3.5 (Storer et al., 2021) by considering all the hits with an e-value < 1e-50 and a bit-score > 50 significant.

## Results

### Improved Annotation of the Cephalopod Repeat Complement

Roughly 40–50% of the total coleoid assembly lengths were masked in the first round, whereas only 30% of the *Nautilus pompilius* genome was masked. An additional 2–6% was uncovered in the second round of the hard-masked genome, highlighting the importance of the second round of genome masking. As a result, the double masking revealed the repeat content to constitute about half of all the genomes considered, except for *Nautilus* (Supplementary Table S1). The double masking has been proven to be a useful approach for capturing huge amounts of repetitive DNA in noticeably big genomes, such as that of *Neoceratodus forsteri* (Meyer et al., 2021). In our case, cephalopod genomes are around 10-fold smaller and less repetitive than the Australian lungfish genome. Even so, TE annotation was enhanced in terms of both sequence quantity and number of detected families; for instance, the second masking round allowed to identify SINEs in *E. scolopes*, which were completely unannotated after just one round. The RepeatCraft step was then able to merge from a minimum of about 53,000 repeat copies in *O. vulgaris* to a maximum of 152,000 in *E. scolopes* (Supplementary Table S1), allowing for the reconstruction of degenerated and fragmented elements.

### Total TE Composition and Activity of TEs in Cephalopod Genomes

**FIGURE 1:**
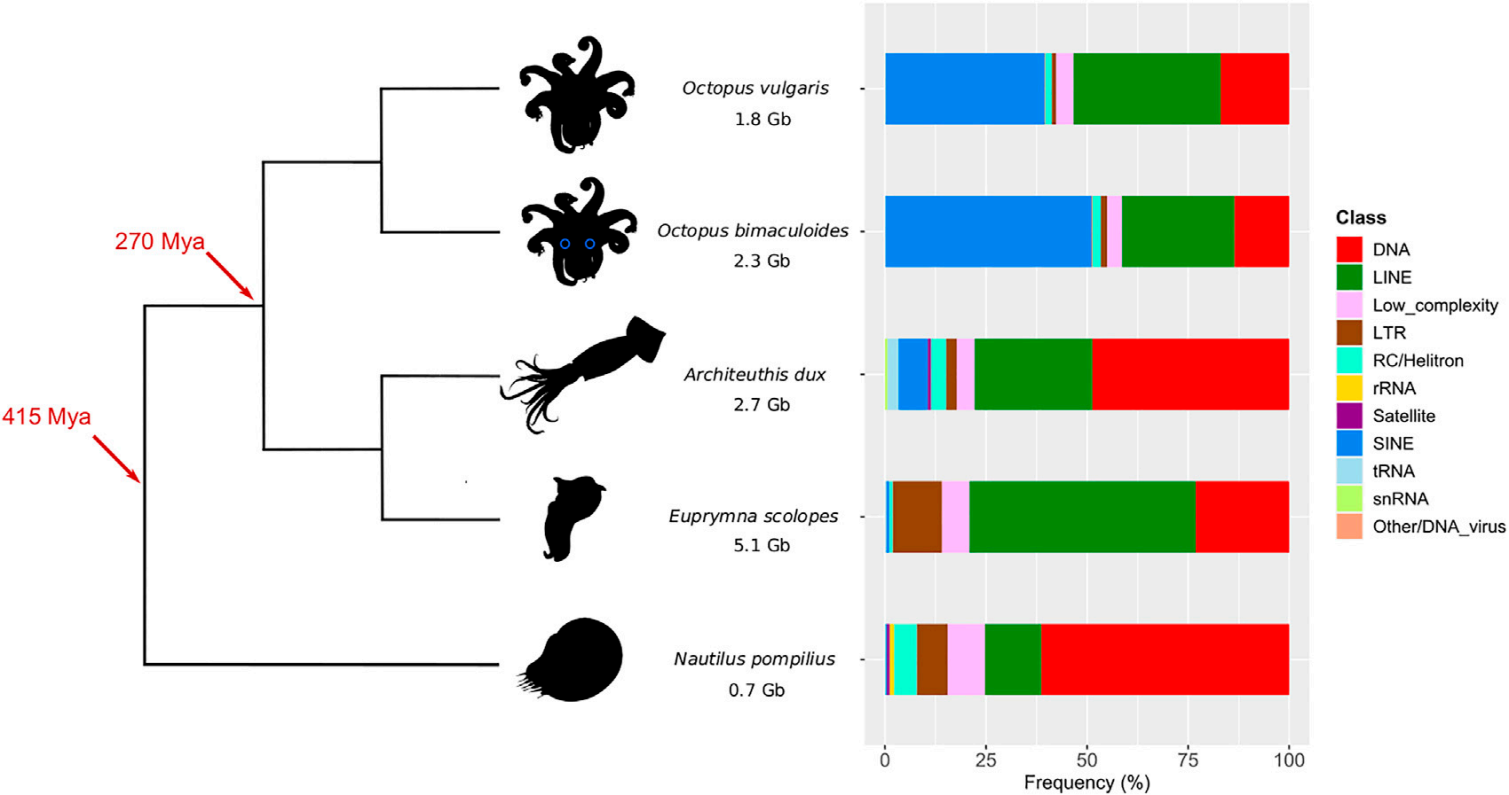
Phylogeny of *O. vulgaris* (common octopus), *O. bimaculoides* (California two-spot octopus), *A. dux* (giant squid), *E. scolopes* (Hawaiian bobtail squid), and *N. pompilius* (chambered nautilus) with their corresponding assembly size and total content of repeat subclasses. Time marks on the tree refer to the coleoid last common ancestor (270 Mya) and the split of the nautiloid outgroup (415 Mya) based on Tanner et al. (2017). Percentages in the barplot are calculated as subclass counts out of the total number of elements in every assembly (unknown and simple repeats are excluded).

As shown in Figure 1, octopus TE subclass compositions are similar between each other, with a major SINE (∼40%) and LINE portion (∼30%), followed by DNA elements (∼17%). Decapodiformes show instead a different landscape: *E. scolopes* features mostly LINEs (56%) and secondly DNA (23%) and LTR subclasses (12%), while SINEs are very scarcely represented (<1%); *A. dux* repeat content mainly consists of DNA elements (49%) and LINEs (29%). Despite having a much more restrained genome (see Supplementary Table S1), the *Nautilus* repeatome is similar to the giant squid one in that the first major subclass is DNA (61%) and the second one is LINE (14%). At the TE family level, tRNA-Core and tRNA-Deu are the main contributors to the octopus-like SINE complement; in *E. scolopes*, LINEs and LTRs are mainly represented by CR1 (29%) and Gypsy elements (11%), respectively. Both *A. dux* and *Nautilus* DNA repeat contents are not defined by one prevailing family but by diverse ones, such as TcMar-Mariner, hAT-Charlie, TcMar-Tc1, hAT-Tip100, and TcMar-Tigger, which also contribute to the DNA element content of the other species (Supplementary Figure S1, Supplementary Table S2).

Overall, the portion of the repetitive genome and subclass composition of each species are consistent with the literature (Albertin et al., 2015; Belcaid et al., 2019; Zarrella et al., 2019; Fonseca et al., 2020; Zhang et al., 2021 (see Discussion for details). The mapping of transcriptomic data against genomes and calculating their overlap with repeat annotations revealed the proportion of elements expressed in at least one of the sampled tissues. A substantial proportion of repeat loci showed putative expression. While we found large differences in the proportion of loci with at least one transcriptomic read, with *O. vulgaris* having the lowest (39%) and *N. pompilius* having the highest (92%), this is likely a result of the underlying assembly quality. Moreover, the counts for each repeat category vary between tissues, which may be a result of tissue-specific TE activity within a single organism (Supplementary Table S3).

### Divergence Decomposition Reveals an Ancient Repeat Subset

**FIGURE 2:**
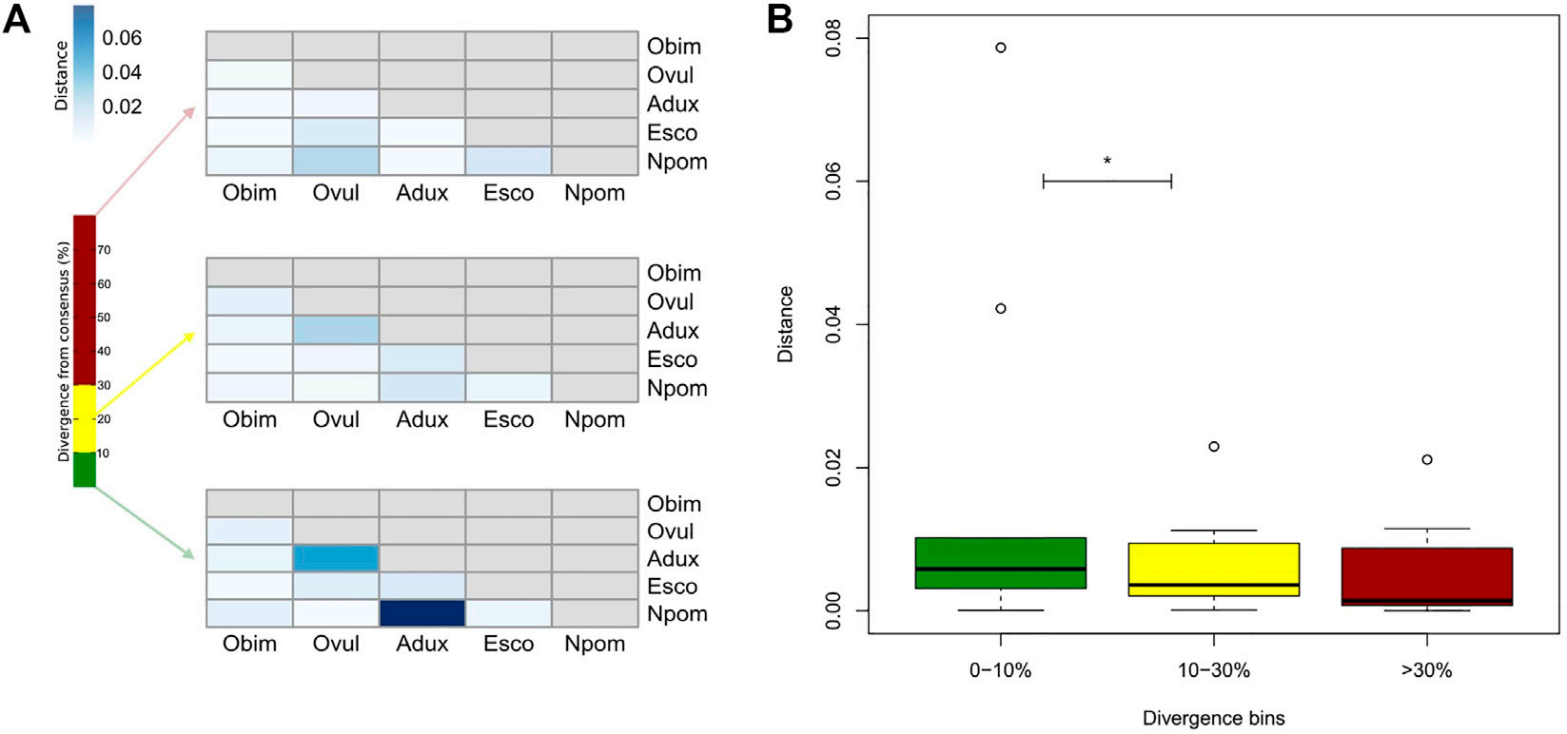
**(A)** Euclidean distances between species according to divergence from consensus of their repeats. Distance (quantitative variable in blue) is calculated on the normalized raw frequencies of just TE families shared by all species in each divergence bin (qualitative variable in percentage). Divergence bins are defined as 0–10% (green), 10–30% (yellow), and >30% (red) ranges. **(B)** The same quantitative distance values for the same qualitative divergence bins are displayed in a boxplot. Black horizontal lines correspond to medians, boxes’ lower and upper ends respectively to the first and third quartiles, whiskers’ lower and upper ends respectively to the minimum and maximum values, and empty circles to the outlier distance values. Asterisks indicate *p* < 0.05 for the Wilcoxon test calculated between the respective distance sets. Adux = *A. dux*; Esco = *E. scolopes*; Npom = *N. pompilius*; Obim = *O. bimaculoides*; Ovul = *O. vulgaris*.

**FIGURE 3:**
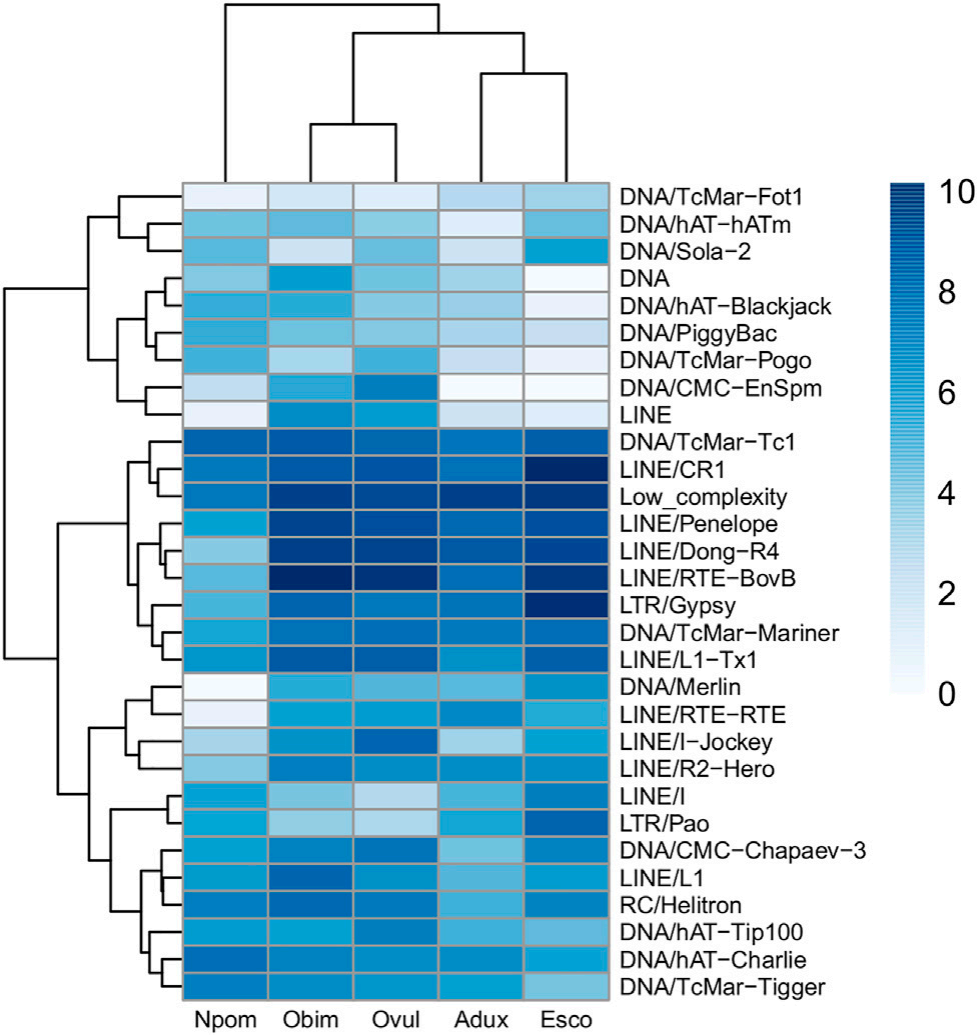
Anciently shared repeat complement obtained from TE families retained by all species at >30% divergence values. TE families are clustered with the ‘complete’ method of the pheatmap package, and species are clustered according to the phylogeny in Figure 1. Log-scaled values of raw element counts are shown.

**FIGURE 4:**
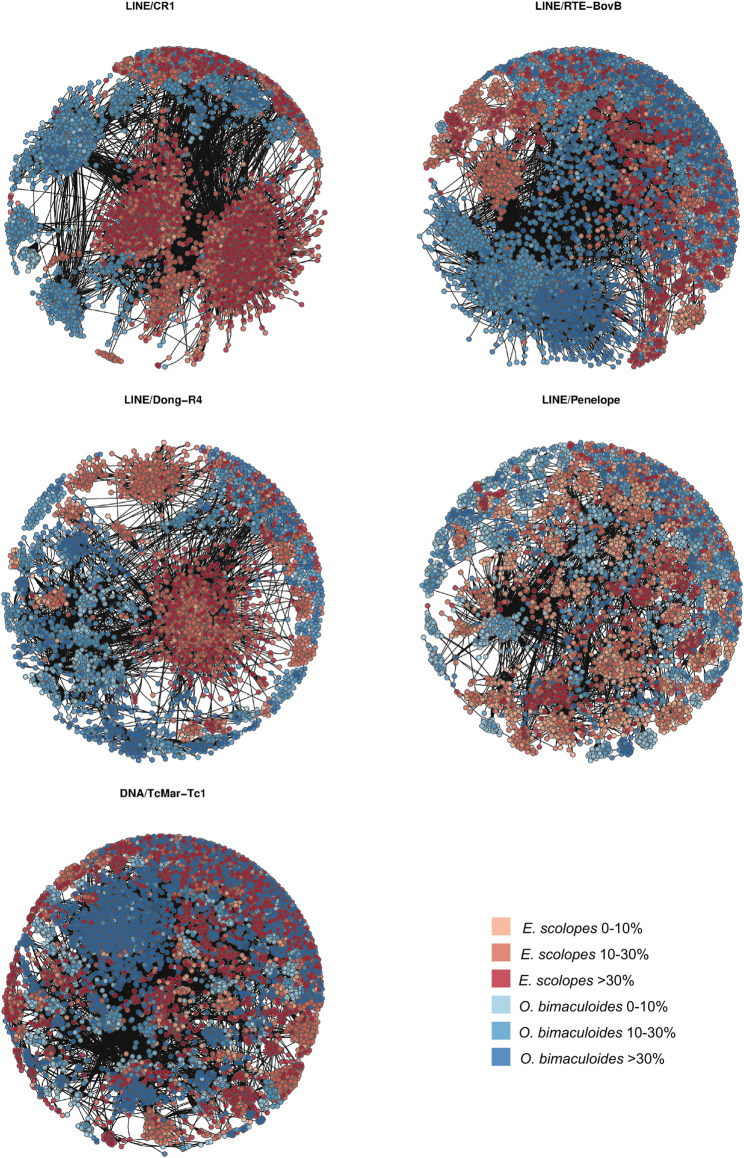
Sequence similarity-based net plots for LINEs CR1, RTE-BovB, Dong-R4, Penelope, and DNA/TcMar-Tc1. Each point corresponds to one TE copy, whose color and shade correspond to a given species and divergence range as per legend and whose position depends on the ratio number of mismatches/alignment length assigned by BLAST.

We find only a slight decrease in the transcriptional activity of older element loci (>30% divergence) in *E. scolopes* and *O. vulgaris* compared to the younger age categories, both overall and at the tissue level (Supplementary Table S3). 0–10 and 10–30% divergence complements are in general more abundant in the genome than in the >30% subset for both the number of TE families in at least one genome and the maximum raw count for a family in a given assembly. Lineage-specific expansions such as those of tRNA-Core, tRNA-Deu, and CR1 recur throughout all the bins as well as some more abundant elements shared by all species, such as LINEs Penelope, Dong-R4 and RTE-BovB, and the DNA elements Mariner and Tc1 (Supplementary Figure S1). Interspecies distances calculated on both shared families and all families are higher in the 0–10% divergence complement and tend to lower as the divergence increases (Figure 2; Supplementary Figure S2). The highest distances are those of *A. dux* against *Nautilus* and *O. vulgaris* and are generally consistent with the differences in repeat family abundance and weights on principal components (PCs) in each bin (Supplementary Figures S1, S3). The extracted anciently shared repeat complement is formed by 15 DNA families, 11 LINEs, 2 LTRs, and Helitrons, (plus tRNA and low-complexity elements) (Figure 3). Almost all families show vastly different genomic abundances across species: in particular, CR1 and Gypsy elements stand out in *E. scolopes*, just as RTE-BovB does in octopuses. Moreover, a specific subset composed of LINEs RTE-BovB, Dong-R4, Penelope, L1-Tx1, CR1, LTR/Gypsy, and TcMar-Tc1 and Mariner DNA elements is expanded in three coleoids, while *Nautilus* and *Architeuthis* show significantly lower copy numbers (*p*-Wilcoxon < 0.05). Although SINEs are very abundant in octopuses, they are underrepresented in Decapodiformes and completely missing from this common ancient coleoid cephalopod repeat set. Raw abundance counts per chromosome of sequences at all divergence levels have linear relationships with chromosome sizes (Supplementary Figure S4). Consistent with the previous observations of possible lineage-specific expansions, the BLAST analysis revealed at least two LINE CR1 bursts in the *E. scolopes* genome and just as many RTE-BovB expansions in the *O. bimaculoides* genome. We also identify smaller expansions of LINE families Dong-R4 and Penelope and DNA/TcMar-Tc1 as octopus- and Hawaiian bobtail squid-specific. Furthermore, the sequence similarity search highlights considerable octopus-squid copy co-groupings for all the families considered (Figure 4). Despite the effort made to make inter- and intraspecies sequence hit proportions as balanced as possible, exactly even retention of both in the search output was not reached (Supplementary Figure S5). The possibility that the marked bias favoring same-species matches could affect to some extent the net plot arrangement should be taken into consideration. The research in Dfam gave significant hits for 12 DNA and 3 LINE families, with TcMar-Tc1, Mariner, and Tigger having the highest number of hits in the database and *A. dux* being the species with the highest number of overall matches (Supplementary Table S4).

## Discussion

### The Repeat Landscape of Cephalopods

By considering five cephalopod species as a proxy of the present diversity, we were able to integrate a common repeat annotation of the available representatives of this clade and to identify the diverging expansion histories that characterize each lineage. Our results at the subclass level are strongly consistent with the literature, and our annotations at the TE family level add valuable knowledge in the context of cephalopod genome architectures. The discrepancy in the number of active elements (as inferred by RNA-seq mapping) across species could be correlated with genome assembly quality. It is worth noting that the *Nautilus* genome, which has the highest proportion of active repeats, is also the only gapless assembly and the one with the highest alignment score of RNA-seq reads. In addition to the technical limitations of fragmented genomes, another reason could be an actual stronger inhibition of transcription that might be at play in genomes more extensively colonized by selfish elements. Despite these factors, the results reported in Supplementary Table S3 are consistent with the expression of a substantial portion of the annotated repeatomes.

### Sequence Divergence Decomposition Accounts for Different Phylogenetic Signal Between Species

The general trend of lower genomic counts above the 30% divergence level measured from the repeat consensus is due to the decreasing ability of RepeatMasker to find repeats as their divergence to the consensus increases as well as many ancient sequences being lost from the genome. However, we consider the recurrence of a given TE set in the highest divergence bin of all species as a strong signal of TE basal retention across coleoids and some in their outgroup. In support of this, interspecies distances are on the whole higher in the 0–10% interval and progressively lower in the 10–30% and >30% intervals (Figure 2, Supplementary Figure S2). Repeat composition at different divergence windows can thus be accounted for with a good approximation for more recent or ancestral scenarios: 0–10% complements tend to mirror specific novel TE bursts or new family emergence, causing more marked differences; conversely, >30% divergence contents should consist of conserved families which make species more akin to each other. TE activity patterns can significantly vary among lineages, even in the case of a recent evolutionary split (Boulesteix et al., 2006), meaning that the comparison of TE content does not necessarily reflect species phylogeny. In our observation, distance results calculated considering all families were similar to those based only on shared ancient families. 0–10% divergence-based PCA places species according to phylogeny along PC1, and among TE families mainly responsible for differences are LINE/RTE-BovB and CR1, the main ones subject to differential expansions (Supplementary Figure S3). Moreover, as divergence increases, octopuses generally cluster together, while *Nautilus* tends to move closer to Decapodiformes, especially *A. dux*, consistently with the different repeat expansion patterns highlighted in the ancient repeat complement (see next paragraph).

### The Ancestral Coleoid Repeat Complement: TE Subclass Composition Insights From the Comparison Across Species

The anciently shared repeat complement obtained primarily consists of LINEs, DNA elements, and one LTR family. SINEs are not present, as reflected in their low counts in *E. scolopes* and *Nautilus*. The considerable length of the *E. scolopes* genome (5.1 Gb) combined with the difficulty in sequencing short interspersed elements could have misled SINE representation. Nevertheless, as suggested by Albertin et al. (2015) and considering the lack of SINE enrichments in other Decapodiformes, the SINEs that we were able to recover likely constitute expansions specific to octopuses. It is important to note that the ancient repeat set shared across *coleoid* species does include some SINE families (Supplementary Figure S1), suggesting that these retroelements could have been active in the genome of their common ancestor. The slow evolutionary rate and the repeat content found in the *Nautilus* genome by Zhang et al. (2021) might suggest the retention of signatures similar to those of the pre-radiating coleoid ancestor. Therefore, the fact that *Nautilus* generally lacks highly divergent SINEs points to their actual absence in the ancient repeat complement of cephalopods. Whether and to what extent SINEs also initially contributed to the ancestral cephalopod genome remain unclear due to SINE fast evolution and sequence decay that may have occurred during more than 270 million years. As shown by Supplementary Figure S3, *Nautilus* and *A. dux* cluster separately from the other species because of the weaker genomic expansion of their shared complement, especially LINEs and LTRs; DNA elements instead display more restrained expansion patterns in all species (Figure 3). Assuming that these TE subclasses were all present in the common ancestor, this suggests the cephalopod and molluscan plesiomorphic and conserved nature of the DNA transposon complement and the dynamic nature and more recent activity of some LINEs that expanded in the coleoid ancestor.

### Chromosomal Distribution and Expansion Patterns of Anciently Shared TE Families

The most enriched families emerging in the ancient complement are LINEs Penelope, Dong-R4, CR1, L1-Tx1, L2, RTE-BovB, and DNA/TcMar-Tc1, as well as LTR/Gypsy. Among them, as already mentioned, CR1, RTE-BovB, and Gypsy elements show clear lineage-specific expansions. The linear relationships of element count against chromosome size revealed that TE families belonging to the ancestral complement are not arranged into any chromosomal hotspots in *E. scolopes*: the pattern is the same for both sequences close to and divergent from consensus, meaning that both recent and older TE outbreaks did not occur in specific chromosomes in this species. However, this remains to be verified in other species and does not rule out possible enrichments at finer scales and linked to different terms such as Gene Ontology (GO) or cephalopod-specific synteny (gene order) loci. The scattered distribution of TEs across the genome of *E. scolopes* agrees, however, with the scenario of the extensive and long-standing reshuffling that has arisen in coleoid genomes (Albertin and Simakov, 2020). Additionally, the directly proportional contribution of TEs to chromosome lengths is consistent with the hypothesis that genome size is directly influenced by repetitive DNA (Kidwell, 2002; Naville et al., 2019).

The fact that repeat sets that we deem as apomorphic are still included in the ancient complement stresses the limit of sequence divergence-based methods as we are not able to clearly isolate actual ancestral repeat subgroups. Notwithstanding, the network-based approach identifies clusterings that do not conform with the divergence bins we defined, as both independent outbursts and interspecies groupings appear to consist of all divergence values (Figure 4). This might be a valuable approach for discriminating between recently proliferated elements and the more interspecies connected ancestral and conserved copies that are putative remnants of the ancient expansions. The common octopus-squid clusters could thus be informative in revealing ancient repeats across such divergent lineages, potentially pointing to conserved TE subsets in coleoids.

Although the similarity networks and our Dfam similarity analysis suggest that repeat bursts occurred through vertical transmission, we cannot rule out occasional horizontal transfer events for more ancient elements. While we did not find evidence for homology across long-diverged taxa for CR1, Penelope, and Dong-R4, hits were obtained for RTE-BovB and TcMar-Tc1 (in addition to other DNA elements), mostly corresponding to aquatic vertebrates. Nevertheless, most of these species were the most closely related to cephalopods in Dfam. The origin of these repeat elements in cephalopods is therefore equally likely via vertical transmission.

### Conclusion and Next Steps

The family repeat content was outlined in five cephalopod species, and a preliminary assessment of an ancestral TE set was made by considering the most divergent repeat sequences. This allowed us to distinguish between lineage-specific, shared, and stem-coleoid expanded repeat elements. An additional sequence similarity-based analysis of some ancestrally shared families revealed more accurate patterns of independent and interspecies expansions, therefore highlighting a possible partially shared history of such repeat families. The comparative profiling here described is preliminary work, and the inclusion of new key species and chromosome-level data will be essential for making the coleoid and cephalopod TE landscape more robust. Indeed, the recent genome sequencing of *Nautilus* added an important comparative point to our study as the only coleoid outgroup, and future acquisition of new data regarding nautiloids and new coleoid species will be fundamental for investigating the cephalopod repeatome evolution. Similarly, further studies such as gene ontology enrichment, orthology construction, and synteny breakage enrichment could shed light on whether the TE subgroups obtained with our method were actually involved in cephalopod genome reshuffling and to test our approach to track down the repeat complement of the early (coleoid) cephalopods.

## Data Availability

Publicly available datasets were analyzed in this study. These data can be found here: https://www.ncbi.nlm.nih.gov/assembly/GCA_018389105.1/ GenBank assembly accession: GCA_018389105.1, https://www.ncbi.nlm.nih.gov/assembly/GCA_003957725.1/ GenBank assembly accession: GCA_003957725.1, https://www.ncbi.nlm.nih.gov/assembly/GCF_001194135.1/ GenBank assembly accession: GCA_001194135.1, https://www.ncbi.nlm.nih.gov/assembly/GCA_006491835.1/ GenBank assembly accession: GCA_006491835.1, http://metazoa.csb.univie.ac.at/data/v2/, https://www.ncbi.nlm.nih.gov/sra/?term=SRR2047118 SRA accession: SRR2047118, https://www.ncbi.nlm.nih.gov/sra/?term=SRR7645642 SRA accession: SRR7645642, https://www.ncbi.nlm.nih.gov/sra/?term=SRR2047116 SRA accession: SRR2047116, https://www.ncbi.nlm.nih.gov/sra/?term=SRR2047109 SRA accession: SRR2047109, https://www.ncbi.nlm.nih.gov/sra/?term=SRR2047111 SRA accession: SRR2047111, https://www.ncbi.nlm.nih.gov/sra/?term=SRR2857274 SRA accession: SRR2857274, https://www.ncbi.nlm.nih.gov/sra/?term=SRR7548187 SRA accession: SRR7548187, https://www.ncbi.nlm.nih.gov/sra/?term=SRR13005724 SRA accession: SRR13005724, https://www.ncbi.nlm.nih.gov/sra/?term=SRR8159234 SRA accession: SRR8159234, https://www.ncbi.nlm.nih.gov/sra/?term=SRR8172522 SRA accession: SRR8172522, https://www.ncbi.nlm.nih.gov/sra/?term=SRR3493852 SRA accession: SRR3493852, https://www.ncbi.nlm.nih.gov/sra/?term=SRR2857280 SRA accession: SRR2857280, https://www.ncbi.nlm.nih.gov/sra/?term=SRR13131286 SRA accession: SRR13131286.

## Abbreviations

BLAST, Basic Local Alignment Search Tool; GO, Gene Ontology; HGT, Horizontal Gene Transfer; LINE, Long Interspersed Nuclear Element; LTR, Long Terminal Repeat; PCA, Principal Component Analysis; SINE, Short Interspersed Nuclear Element; TE, Transposable Element.
