# Supplementary material for: Repeat Age Decomposition Informs an Ancient Set of Repeats Associated With Coleoid Cephalopod Divergence
Source: Front Genet. 2022 Mar 14;13:793734. doi: 10.3389/fgene.2022.793734 (PMC8967140; doi:10.3389/fgene.2022.793734)
Supplement: Supplementary file 1 [file DataSheet2.pdf]

Supplementary Material

Supplementary Figures

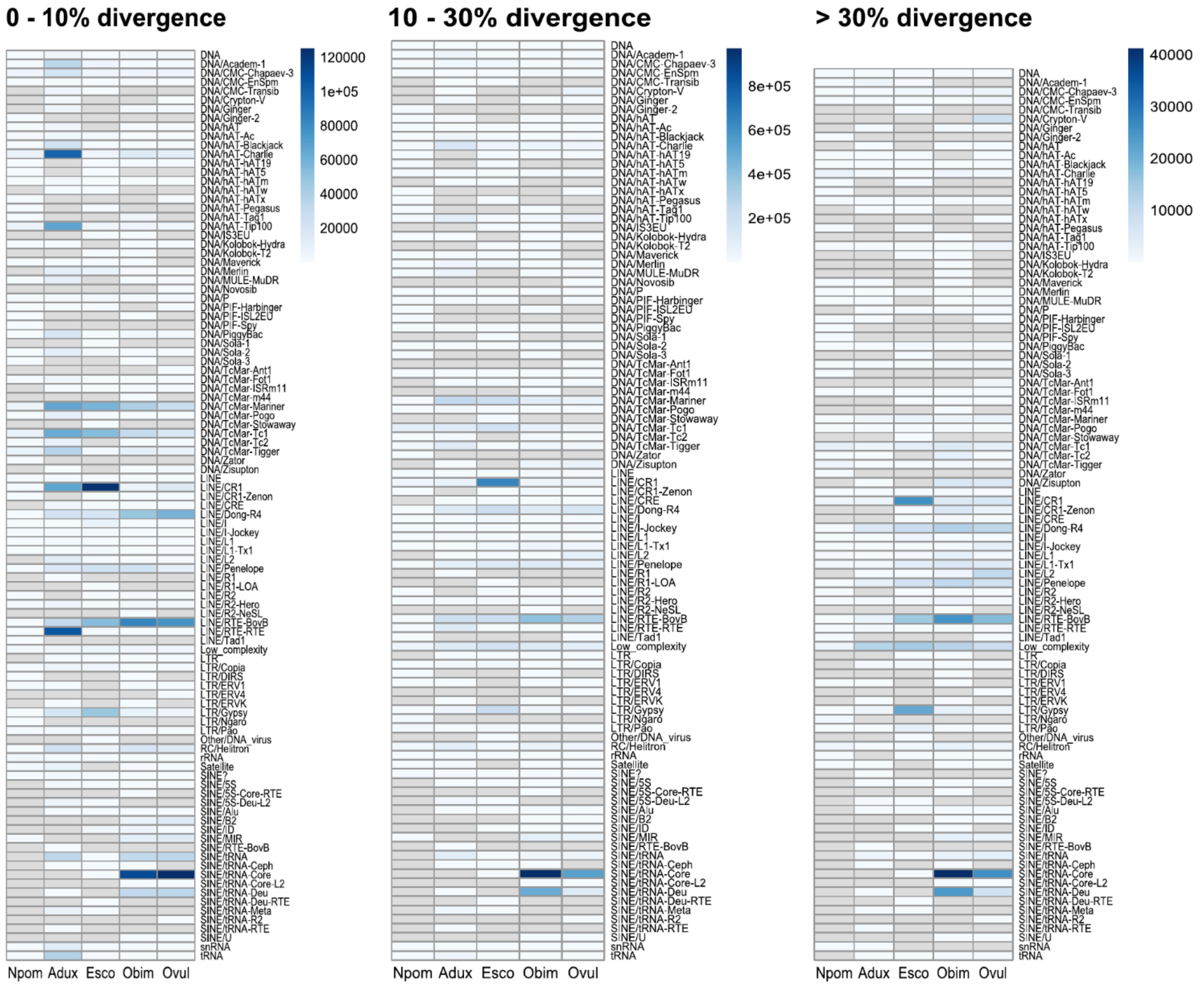

Supplementary Figure S1. Complete TE family composition of every species divided into 0-10%, 10-30% and >30% divergence bins. The scale refers to raw element counts; grey cells indicate a missing family. Adux=*A. dux*; Esco=*E. scolopes*; Npom=*N. pompilius*; Obim=*O. bimaculoides*; Ovul=*O. vulgaris*.

(A) **(B)**

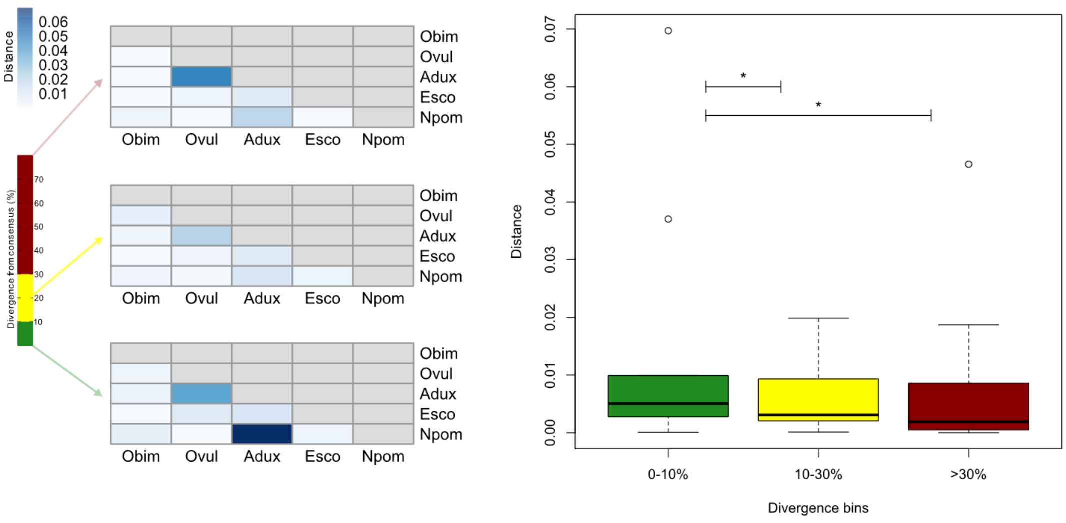

Supplementary Figure S2. Euclidean distances between species according to divergence from consensus of their repeats. **(A)** Distance (quantitative variable in blue) is calculated on the normalized raw frequencies of all TE families presented in Figure S1 divided by divergence bin (qualitative variable in percentage). Divergence bins are defined as 0-10% (green), 10-30% (yellow) and >30% (red) ranges. **(B)** The same quantitative distance values for the same qualitative divergence bins are displayed in a boxplot. Black horizontal lines correspond to medians, boxes' lower and upper ends respectively to the first and third quartile, whiskers' lower and upper ends respectively to the minimum and maximum values, and empty circles to the outlier distance values. Asterisks indicate $p < 0.05$ for Wilcoxon test calculated between the respective distance sets. Adux=*A. dux*; Esco=*E. scolopes*; Npom=*N. pompilius*; Obim=*O. bimaculoides*; Ovul=*O. vulgaris*.

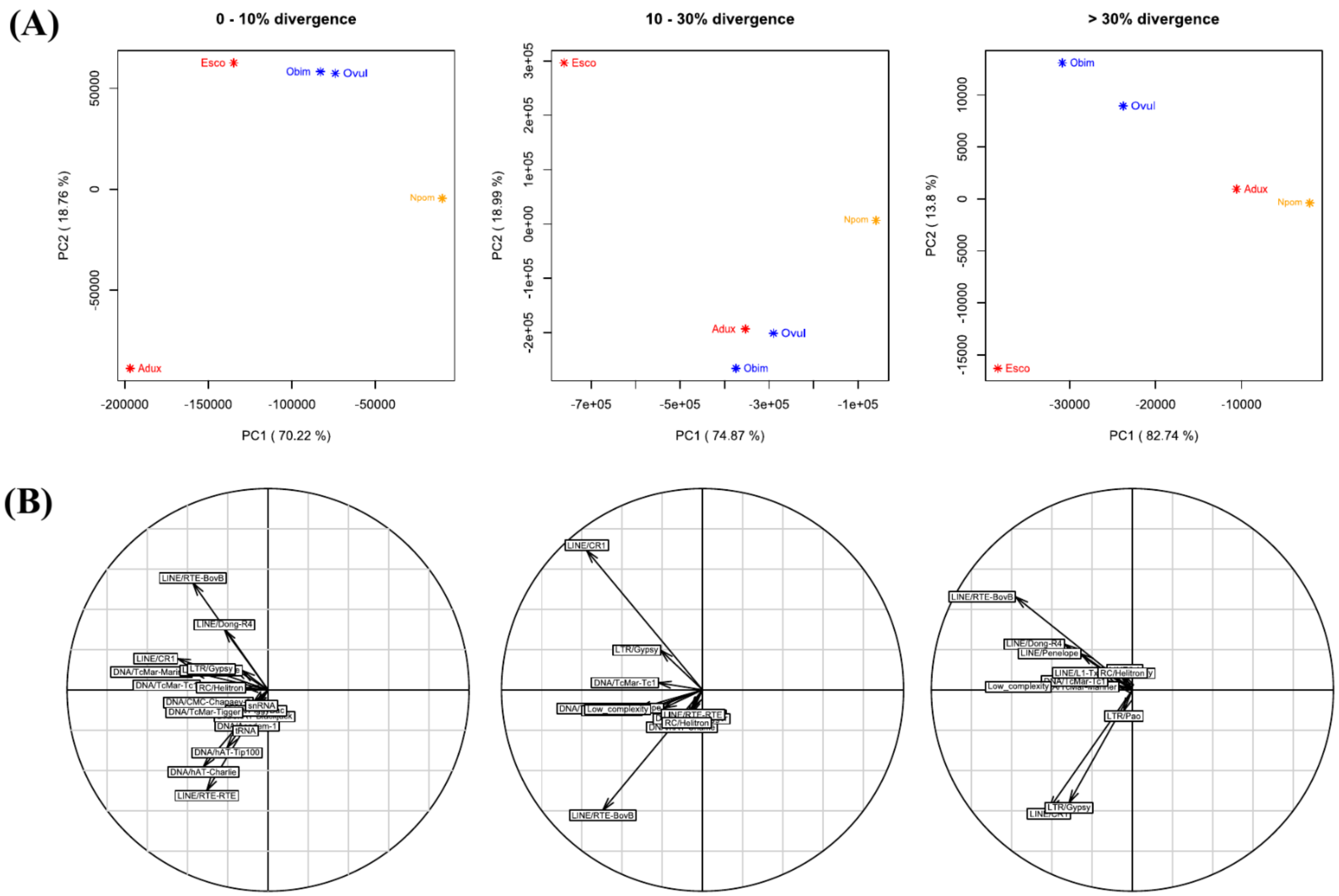

Supplementary Figure S3. Principal Component Analysis (PCA) calculated on only TE families shared across species **(A)** and corresponding loadings **(B)** in 0-10%, 10-30% and >30% divergence ranges. Species scores and loadings of original variables having correlation coefficient higher than 0.5 or lower than -0.5 are shown along PC1 and PC2.

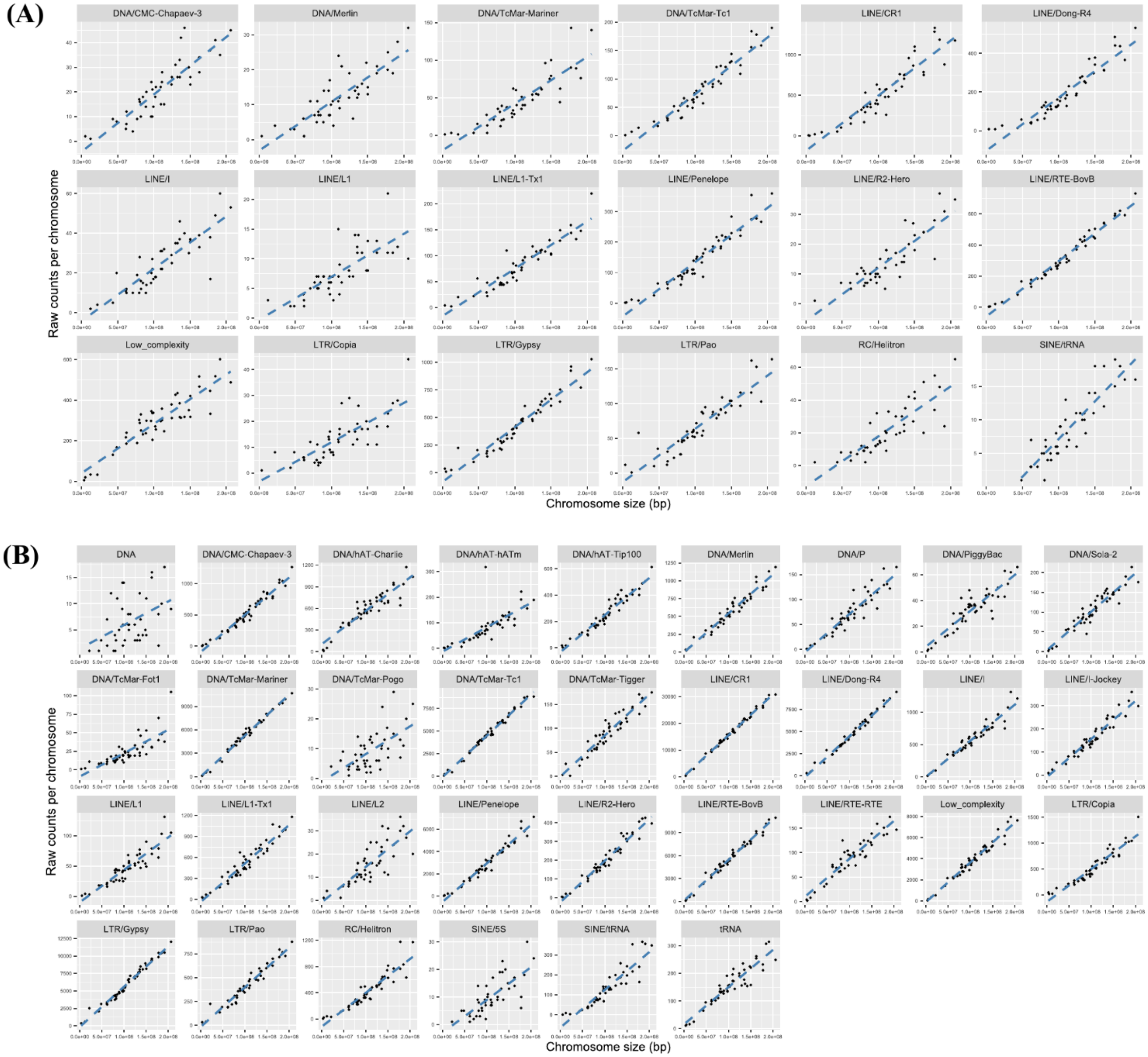

Supplementary Figure S4. TE family raw counts per chromosome against chromosome lengths fitted to a linear model in *E. scolopes*. Families shown are the ones belonging to the ancient repeat complement and whose total sequences number was higher than 300. Both repeats from solely the >30% divergence bin **(A)** and total content **(B)** are shown for each TE family.

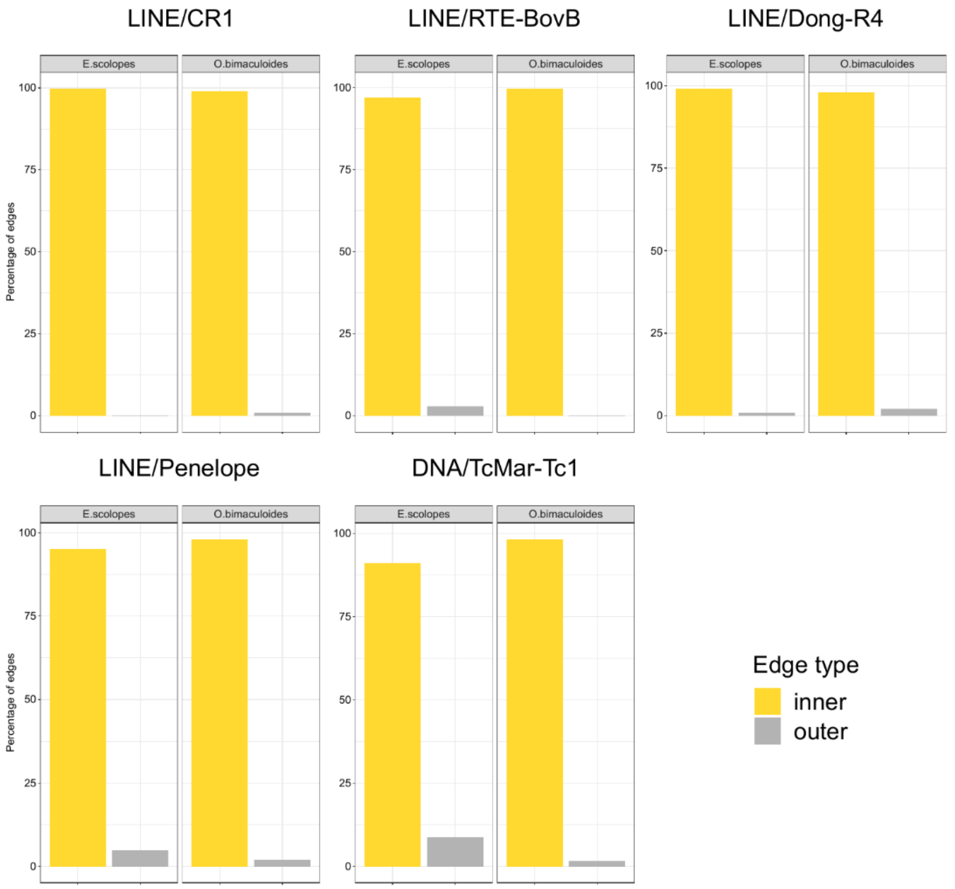

Supplementary Figure S5. Percentages of inner and outer nodes of *E. scolopes* and *O. bimaculoides* sequence similarity analysis for CR1, RTE-BovB, Dong-R4 and Penelope LINE families, and DNA/TcMar-Tc1 family. Inner nodes (yellow) represent within species hits, outer nodes (grey) correspond to inter-species matches.
